# Obstetrics and Gynecology

**Published:** 1894-12

**Authors:** 


### OBSTETRICS AND GYNECOLOGY.

EDITED BY DR. VIRGIL O. HARDON.

### PUERPERAL SEPSIS.

In an article on this subject, *Buffalo Medical and Surgical Reporter*, Dr. William Warren Potter offers the following conclusions:

1. Obstetric engagements once accepted should be faithfully fulfilled, no matter how awkwardly they fit. Apply the same rule of cleanliness to rich and poor alike. Decline service when this cannot be done. Human life is too precious to jeopardize it by slipshod, half-hearted, or indifferent service.

2. The physician should be a model of cleanliness in body and clothing, and should insist upon the observance of similar conditions by all persons in and about the lying-in chamber.

3. The delivery room, whether in hovel or palace, court, alley, or avenue, should be simple in its furniture and hangings, and be cleaned with soap, water, and whitewash (if possible to use the latter) immediately before occupancy by the puerpera.

4. The delivery bed should consist of a new tick, filled with sweet and clean straw, covered with a blanket, impervious dressing, and a folded sheet, with other clean covering to be allowed, according to season. Exceptions to this simple bed should be as few as possible, and in no event should a bed be substituted that has been used by the sick, or that is not beyond even a suspicion of infection.

5. The patient should be specially prepared for delivery by baths and enemata, vaginal douches, and clean clothing; and labor should be conducted on the lines of absolute cleanliness, with a few digital examinations, and a complete delivery of the secundines.

6. Lesions of the genital tract should receive careful attention; rents of the perineum should be repaired, and so, too, in some instances, should tears of the cervix.

7. Antiseptic solutions containing a germicide should be used for cleaning the hands and instruments of the operator. Intra-uterine irrigation with sterilized water should be carefully employed after operative midwifery, either manual or instrumental.

8. Finally, if sepsis proceed to suppuration and abscess, the abdomen should be opened, pus cavities emptied, irrigation used, and drainage established. If the uterus and adnexa become thoroughly infected, they should be extirpated.—*Canada Medical Record.*

---

### THE TREATMENT OF CYSTITIS IN THE FEMALE.

The treatment of acute and chronic cystitis in the female is clearly dealt with by A. LUTAUD (*Journal de Médecine de Paris*, July 22, 1894).

*Acute Cystitis.*—In this form the first indication is to combat pain. The author recommends the following suppository:

| | |
|---|---|
| Morphine hydrochlorate, | |
| Cocaine hydrochlorate, of each | gr. 1-6. |
| Extract of belladonna | gr. 1-12. |
| Cacao butter | grs. 46. |

This suppository is introduced every four hours, until the pain and tenesmus cease. For insomnia this rectal injection is advised:

| | |
|---|---|
| Chloral hydrate | grs. 60. |
| The yolk of an egg. | |
| Water or milk | ʒ 4¾. |

For general treatment, hot fomentations, poultices, and sitz-baths are recommended, as is also the introduction into the vagina, morning and evening, of the following tampon:

| | |
|---|---|
| Camphorated lanolin | grs. 460. |
| Extract of belladonna | grs. 30. |

*Chronic Cystitis.*—When the pain and inflammation have subsided, an elastic or glass catheter, to which is attached a syringe holding from 3¼ to 4¾ ounces, is introduced into the bladder and the following injection put in:

| | |
|---|---|
| Boric acid | ℥ 1¼. |
| Biborate of sodium | ʒ 1¼. |
| Distilled water | pts. 1.76. |

Of this solution from 1 to 1½ ounces, according to the irritability of the bladder, are injected. This is followed by the injection of 4¾ ounces of warm water holding in solution the following mixture:

| | |
|---|---|
| Powdered iodoform | grs. 462. |
| Glycerin | ℥ 1¼. |
| Distilled water | ʒ 5. |
| Gum tragacanth | grs. 4. |

Buchu and pichi are of service in the general treatment, and if pus exists in the urine, the following prescription is advised:

| | |
|---|---|
| Benzoic acid | grs. 16. |
| Orange-flower water | ℥ 1½. |
| Boiled water | ℥ 30. |
| Sugar | ℥ 3¼. |

Of this a glassful is to be taken between meals.

Of course, the treatment must be modified according to individual cases.—*Therapeutic Gazette.*

---

### VOMITING IN PREGNANCY.

Lutaud (*Rev. Obstet. et Gyn.*) states that vomiting of pregnancy is best treated by cocaine. The action of this drug is often strengthened by combining it with antipyrin. Thus the following prescription:

| | |
|---|---|
| Chlorhyd. cocaine | gr. iss. |
| Antipyrin | gr. xvi. |
| Aq. dest | ℥ iv. |

Sig.—Teaspoonful every half-hour until the vomiting ceases.

If the stomach will not tolerate this quantity of liquid, ten drops of a one and a half or two per cent. solution of cocaine are administered, repeated at one or two-hour intervals.

At times the application of cocaine to the os is extremely valuable. The following prescription may be used:

| | |
|---|---|
| Hydrochlor. cocaine | gr. xvi. |
| Ext. bellad | gr. iv. |
| Vaseline | ℥ ss. |

Cotin's method of dilating the os with the finger sometimes causes immediate cessation of vomiting. Occasional success will follow Routh's procedure, which consists in exposing the uterine neck by means of a speculum and painting with tincture of iodine. In cases of moderate severity the following mixture will be found serviceable:

| | |
|---|---|
| Tinct. iodine<br>Chloroform | āā ʒ ii. |

Sig.—Five drops night and morning at meal times, taken in Seltzer-water.—*Kan. Med. Record.*

